# Insight into the relationship between aryl-hydrocarbon receptor and β-catenin in human colon cancer cells

**DOI:** 10.1371/journal.pone.0224613

**Published:** 2019-11-01

**Authors:** Kazuhiro Shiizaki, Kenta Kido, Yasuhiro Mizuta

**Affiliations:** Department of Applied Biosciences, Faculty of Life Sciences, Toyo University, Itakura-machi, Oura-gun, Gunma, Japan; Augusta University, UNITED STATES

## Abstract

β-Catenin is a multi-functional protein involved in cell adhesion and signal transduction and has a critical role in colorectal cancer development. β-Catenin positively regulates the aryl-hydrocarbon receptor (AhR) mediated signal by both induction of AhR expression and enhancement of AhR-dependent gene induction. Conversely, it was reported that AhR negatively regulates the β-catenin signal via ubiquitination and subsequent degradation in a ligand dependent manner. However, there have been conflicting data among previous studies regarding the relationship between these two proteins. In this report, we conducted confirmatory studies dissecting the relationship between AhR and β-catenin. We did not observe β-catenin degradation by AhR ligands in several colon cancer cell lines. Reporter assays revealed that the AhR ligand did not alter TcF/β-catenin dependent transcription. Yeast and mammalian two-hybrid assays failed to reconstruct the interaction of β-catenin and AhR even when other factors, Arnt, CUL4B, and DDB1, were co-expressed additionally. Independently to induction of AhR expression, β-catenin enhanced AhR-dependent transcriptional activation via the xenobiotic response element (XRE). Coimmunoprecipitation detected the formation of a β-catenin and ligand-activated AhR complex, which was thought to reflect the β-catenin mediated enhancement of the AhR signaling. Overall, we could only confirm unidirectional interaction, which is positive regulation of the AhR signal by β-catenin. These results suggested that data from previous reports on the degradation of β-catenin via liganded AhR warrants further investigation to yield clarity in the field.

## Introduction

Wnt family signals are triggered by extracellular glycolipoprotein and mediated by intracellular β-catenin. β-Catenin is a component of the cadherin protein complex that regulates cell–cell adhesion. In the nucleus, β-catenin also acts as a transcriptional coactivator of the T-cell factor and lymphoid enhancer-binding factor 1 (Tcf/Lef) family [1–2]. Intracellular quantity of β-catenin is well regulated by phosphorylation, and subsequent degradation is precisely executed by Axin1, CK1, GSK-3β, and APC. Mutations of these factors as well as β-catenin fail to degrade β-catenin and lead to cytoplasmic accumulation of β-catenin. The cytoplasmic β-catenin translocates into the nuclei, acts as a coactivator of Tcf/Lef family transcription factors, and activates the expression of Wnt/β-catenin target genes, including c-myc, axin2, and cyclin D1. Those genes play important roles in tumorigenesis as well as cell proliferation [3–4].

The aryl-hydrocarbon receptor (AhR) is associated with tumorigenesis in a distinct way from β-catenin [5]. AhR is a transcriptional factor activated by binding to ligands such as 2,3,7,8-tetrachlorodibenzo-p-dioxin (TCDD), benzo[a]pyrene (BaP), and 3-methylcholanthrene (3MC) [6]. The liganded AhR translocates into the nucleus and binds a specific sequence with its binding partner, Arnt [7]. The AhR/Arnt heterodimer complex binds XRE (xenobiotic responsive element) sequences in the promoter regions of target genes and regulates their expression [8]. The target genes, such as genes encoding CYP1 family enzymes, of AhR are involved in xenobiotic metabolism. These gene products can metabolically activate numerous pre-carcinogens, including AhR ligands such as 3MC and B[a]P. Therefore, AhR is considered as one of the mediators in tumorigenesis due to activation of pre-carcinogens. Actually, BaP carcinogenicity is lost in the skin of AhR knockout mice [9]. AhR is an aggravating factor for tumorigenesis [5].

Interestingly, numerous studies have reported on crosstalk of β-catenin and AhR [10]. It has been shown that AhR is a target gene of the Wnt/β-catenin signaling in prostate cancer cells [11]. Via transcriptome analysis, an overexpressed hyperactive form of β-catenin (aa 24–47 deleted) was identified to induce AhR gene transcription. Furthermore, the reporter vectors containing dioxin-responsive elements were activated by functional S33Y-mutated β-catenin [12]. β-Catenin was shown to translocate to the nucleus via amino acid substitution or LiCl treatment and subsequently enhanced AhR-mediated CYP1A1 induction [13]. These reports indicated that there must be positive crosstalk between β-catenin signaling and AhR function. Moreover, an interaction in the reverse direction was also found. Ohtake and colleagues reported AhR functions as a component of the E3 ubiquitin ligase to degrade estrogen receptors and androgen receptors [14–15]. For ubiquitin ligase activity, AhR directly binds to cullin 4B (CUL4B) as an adaptor protein, and this Cul4B/AhR complex includes DDB1, Rbx1, and TBL3. AhR also targeted β-catenin as an ubiquitination substrate, and AhR deficient mice have spontaneously developed cecal tumors as the result of β-catenin accumulation [16]. In the report, natural AhR ligands, indole-3-acetate (IAA), suppressed intestinal tumor formation in APC/min+ mouse by degradation of β-catenin. The phenomenon that AhR degrades β-catenin was clearly demonstrated in several colorectal cancer cell lines, DLD-1, HCT116, and SW480. The molecular mechanisms of this AhR-mediated protein degradation have been well characterized. A component of cullin–RING E3 ubiquitin ligases complex, CUL4B plays a key role in the interaction between AhR and substrate protein and mediates proteasomal degradation after ubiquitination of target proteins [15]. Prevented intestinal tumorigenesis was also shown by dietary administration of AhR ligand indole-3-carbinol wherein E3 ubiquitin ligases inhibited Wnt-β-catenin signaling [17]. Another study revealed AhR inhibited Wnt/β-catenin signals indirectly via reduced R-spondin 2/3 expression [18]. By studying zebrafish embryos, an interaction in which Ahr2 represses active β-catenin signaling was indicated. In a previous report, toxicity of 1-azakenpaullone, a β-catenin activator, to zebrafish embryos decreased in the presence of AhR agonist 6-formylindolo[3, 2-b]carbazole [19].

This mutual regulation between AhR and β-catenin looks like a well-crafted feedback loop: β-catenin enhances the AhR signal, and AhR down-regulates β-catenin signal. However, data from other studies indicated several discrepancies regarding the interaction between β-catenin and AhR. In the hepatoma cell line, TCDD failed to alter β-catenin/TcF reporter activity as well as their target genes such as Axin2 and Gpr49 [13]. In rat liver epithelial cell line WB-F344, the activated form of β-catenin was down-regulated by sustained TCDD treatment for 24 h [20]. In this report, β-catenin degradation was considered to be independent to ubiquitin-associated protein degradation, because proteasome inhibitors could not restore that. These diverse results for the AhR-dependent β-catenin degradation seem to be due to tissue specificity. In the colorectal tumor cell line Caco-2, AhR and β-catenin interaction was observed by changing to calcium-deficient medium but not by TCDD treatment [21]. The results illustrating that TCDD did not attenuate β-catenin levels in Caco-2 cells were consistent with another study [22]. As with TCDD, indole-3-acetate did not alter β-catenin levels, and only high concentrations of tryptamine decreased β-catenin protein levels, although cytotoxicity was considered. Administration of kynurenine, which is a tryptophan metabolite and an endogenous AhR agonist, activated β-catenin and proliferation of human colon cancer cell HCT116 and increased tumor growth in mice [23]. In another colon carcinoma cell line, HT-29, β-catenin expression was decreased by kynurenine, while it was increased by 1-methyl-tryptophan, an inhibitor of indoleamine 2, 3 dioxygenase-1, a key enzyme of kynurenine production [24]. When these previous studies are compared, there are inconsistencies and conflicting data as to whether AhR agonists enhance or attenuate β-catenin and its signal. In this study, we conducted confirmatory studies of the interaction between AhR and β-catenin, especially focused on ligand-dependent β-catenin degradation by AhR and CUL4B.

## Materials and methods

### Chemicals

3-Methylcholanthrene (3MC), indole-3-carbinol (I3C), indole-3-acetate (IAA), cycloheximide (CHX), and β-naphthoflavone (βNF) was purchased from FUJIFILM Wako Pure Chemical Corporation (Osaka, Japan). Indirubin was kindly provided by Dr. Tomonari Matsuda (Kyoto University, Kyoto, Japan). MG-132 was purchased from Abcam (Tokyo, Japan). All other chemicals and solvents used were purchased from Sigma-Aldrich (St. Louis, MO, USA). Ligands were dissolved in dimethyl sulfoxide (DMSO) and added to media. The final concentration of DMSO was adjusted to 0.1% (*v/v*) in culture media.

### Plasmid construction

The reporter plasmid pX4TK-Luc, including the firefly luciferase gene under the control of four copies of XRE and the thymidine kinase promoter, was a gift from K. Sogawa (Tohoku University, Sendai, Japan). Both TOPflash and FOPflash plasmids used for the TcF reporter assay were obtained from Millipore (Bedford, MA, USA). The mammalian expression plasmid containing mouse (C57BL) and human AhR cDNAs as well as human Arnt and human SRC-1 cDNA were constructed in our previous study [25]. Human β-catenin, CUL4B, and DDB1 cDNA were amplified by RT-PCR from mRNA extracted from HepG2 or MCF-7 cells. Via PCR, an appropriate restriction enzyme site was added to each amplified cDNA and inserted into the vector. For over-expression in mammalian cells, pCI-neo mammalian expression vector (Clonetech) was used, and for expression in yeast, the pESC-His vector (Stratagene, La Jolla, CA, USA) was used. Vectors for the mammalian two-hybrid assay were included in the CheckMate/Flexi Vector Mammalian Two-Hybrid System (Promega, Madison, WI, USA), and vectors for the yeast two-hybrid assay were included in the Matchmaker Gold Yeast Two-Hybrid System (Clontech, Palo Alto, CA, USA). To generate Ser37Ala (S37A) β-catenin, a constitutively active β-catenin mutant [26], a single point mutation was induced using the QuikChange^TM^ Site-Directed Mutagenesis Kit (Stratagene, La Jolla, CA, USA). Expression plasmids for the AhR subdomain and deleted AhR were constructed using PCR. Supporting information S1 Table shows all primers for plasmid construction.

### Yeast two-hybrid assay

The plasmid vector pGADT7, pGBKT7, and their derivative plasmids were co-expressed in yeast strain Y187. Each yeast strain was cultured overnight at 30°C in synthetic medium lacking tryptophan, uracil, and/or leucine. Then, 5 μL of yeast culture was added to 100 μL of synthetic medium and exposed to 1 μM of 3MC, and the 96-well microtiter plates were incubated for 12 h at 30°C. After incubation, yeast cell suspension (10 μL) was added to 100 μL of Z-buffer (60 mM Na_2_HPO_4_, 40 mM NaH_2_PO_4_, 1 mM MgCl_2_, 10 mM KCl, 2 mM dithiothreitol, and 0.2% sarcosyl, adjusted to pH 7.0) containing 1 mg/ml *o*-nitrophenol-β-D-galactopyranoside and subsequently incubated for 60 min at 37°C. The absorbance at 405 and 595 nm was measured to indicate the amount of generated *o*-nitrophenol and yeast cell density, respectively.

### Cell cultures and transfection

All cell lines used in this study were obtained from the American Type Culture Collection (Rockville, MD, USA). DLD-1 cells were also purchased from the Health Science Research Resources Bank (HSRRB, Osaka, Japan). The human epithelial carcinoma line, HeLa, was grown in MEM containing 10% charcoal-stripped FBS and 1× Antibiotic-Antimycotic (Thermo Fisher Scientific, Carlsbad, CA, USA). Human colon cancer cell lines, Caco-2, DLD-1, HCT116, LS174T, SW480, and WiDr; human hepatoma cell line HepG2; and human breast adenocarcinoma cell line MCF-7 were grown in phenol red free DMEM containing 10% charcoal-stripped FBS and 1× Antibiotic-Antimycotic. All cultures were incubated at 37°C in 5% CO_2_. Transfections were performed via the liposome method. Briefly, a total of 1 μg plasmid DNA and 4 μL Plus reagent (Thermo Fisher Scientific) were combined in 200 μL Opti-MEM (Thermo Fisher Scientific). After incubation for 15 min, 2 μL Lipofectamine 2000 reagent (Thermo Fisher Scientific), diluted with 200 μL Opti-MEM, was added and incubated for an additional 15 min. The cells were plated in 6-well tissue culture plates at 30–40% confluence a day before transfection. Liposomes were added in serum-free medium for 3 h and then replaced with MEM or DMEM containing 10% charcoal-stripped FBS without 1× Antibiotic-Antimycotic. In AhR knockdown experiments, HCT116 cells were transfected with human AhR (Ambion, Austin, TX, USA) or control (Ambion) siRNA at 10 nM in a culture medium using Lipofectamine RNA iMAX (Invitrogen, Waltham, MA, USA).

### Measurement of mRNA by semi-quantitative RT-PCR

Total RNA was isolated using Isogen (Nippon Gene, Tokyo, Japan) according to the manufacturer’s instructions. An aliquot (2 µg) of total RNA was subjected to reverse transcription using SuperScriptIII reverse transcriptase (Thermo Fisher Scientific) and oligo-dT primers. cDNA was diluted and amplified by Ex Taq polymerase (Takara, Kyoto, Japan) with gene-specific primers. Primers for human CYP1A1 cDNA amplification were: forward primer, 5'‐CATAGACACTGATCTGGCTGCAG‐3' and reverse primer, 5'‐GGGAAGGCTCCATCAGCATC‐3'. For semi-quantification of the amplified PCR products, samples were collected every 3 cycles between 24–36 cycles and applied to electrophoresis.

### Luciferase assay

The effects of AhR ligands on TcF-dependent transcription were elucidated by transfection of the TOPflash or FOPflash plasmid with other expression plasmid into cells. The effects of AhR ligands on XRE-dependent transcriptional activity were evaluated by the reporter plasmid pX4TK-Luc [27]. Reporter assays were performed by co-transfecting the reporter plasmid mentioned above with the *Renilla* luciferase (*R*luc) expression vector pRL-CMV (Promega). The transfected cells were washed with cold PBS and lysed in 25 μL 1× passive lysis buffer (Promega). Aliquots (10 μL) of the lysates were transferred to microcentrifuge tubes, and firefly luciferase (Luc^+^) and *Renilla* luciferase (*R*luc) activities were measured using the Dual-Luciferase Reporter Assay System (Promega) in the GloMax 20/20 Single Tube Luminometer (Promega). Transfection and translation efficiencies varied between independent experiments, and the results were normalized by calculating Luc^+^/*R*luc ratios.

### Immunoblotting and immunoprecipitation

To prepare the sample for immunoblotting, TNE buffer (10 mM Tris-HCl pH 7.8, 1% NP-40, 150 mM NaCl, and 1 mM EDTA) was used for cell lysis. Cells were washed with PBS and lysed by adding TNE buffer on ice. Then, cell lysates were sonicated moderately and centrifuged at 4°C for 30 min at 20,000×g. For the fractionation of cell lysates, NE-PER Nuclear and Cytoplasmic Extraction Reagents (Pierce, Rockford, IL, USA) were used. According to the manufacturer's protocol, nuclear or cytoplasmic proteins were extracted. Protein concentration in cell lysates was measured by the Bradford method using a Bio-Rad Protein Assay (Bio-Rad Laboratories, Munich, Germany), and 10- or 20 μg aliquot of each sample was separated on an SDS-polyacrylamide gel. Immunoblotting was performed with an anti-human AhR antibody (SA-210; Biomol GmbH, Hamburg, Germany) or with an anti-β-catenin antibody (610154; BD Biosciences Pharmingen, San Diego, CA, USA). Anti-actin antibody (A2066; Sigma-Aldrich, St. Louis, MO, USA), anti-MEK1/2 (47E6; Cell Signaling Technology, Beverly, MA, USA), and anti-Lamine B (sc-6216; Santa Cruz Biotechnology, Santa Cruz, CA, USA) were also used for control experiments. Signals were detected with the peroxidase-labeled secondary antibody contained in the ECL Plus detection system (GE Healthcare Bio-Sciences, Uppsala, Sweden) or anti-goat IgG (KPL, Gaithersburg, MD, USA). Specific protein bands were visualized by the ECL Plus detection system and imaged using ImageQuant LAS-4000 Chemiluminescence & Fluorescence Imaging System (Fujitsu Life Sciences, Japan).

To prepare samples for coimmunoprecipitation, HCT-116 cells were induced to overexpress AhR and β-catenin via pCI-mAhR and pCI-βcat transfection. Twenty-four hours after transfection, cells were exposed to 1 μM 3MC with or without 100 μM MG-132. After 2 h of incubation, the nuclear or cytoplasmic fraction was isolated using a Dounce homogenizer. Nuclear proteins were extracted using the Nuclear Complex Co-IP Kit (Active Motif, CA, USA) according to the manufacturer’s protocol. The extracted protein was measured via Bradford method, and 100 μg aliquot of each sample was diluted with 400 μL of binding buffer containing 10 mM HEPES (pH 7.9), 1.5 mM MgCl_2_, 10 mM KCl, 0.2 mM EDTA, 0.1% NP-40, and Complete^TM^ Protease Inhibitor Cocktail (Roche Applied Science, Mannheim, Germany). Diluted samples were precleared by adding 25 μL (50% slurry) of protein G Sepharose CL-4B (GE Healthcare Bio-Sciences). After 1 hour incubation at 4°C, the CL-4B beads were removed by centrifugation and filtration, and samples were incubated with antibodies (2.5 μL for anti-AhR antibody and 2 μL for anti-β-catenin antibody) for 2-hours at 4°C with rotation. Then, 25 μL of protein G Sepharose CL-4B were newly added to samples and incubated for another one-hour at 4°C. The beads were washed twice with sample buffer mentioned above and finally eluted with 25 μL of sample buffer containing 100 mM Tris-HCl (pH 7.5), 4% 2-mercaptoethanol, and 2% SDS. Immunoblotting was performed with anti-β-catenin antibody, anti-CUL4B antibody (ab67035; Abcam, MA USA) and anti-Arnt antibody (A0972, ABclonal, MA, USA).

### Statistical analysis

Student’s t test was used to evaluate significant differences between two groups.

## Results

### AhR ligands did not reduce β-catenin protein levels

**Fig 1:**
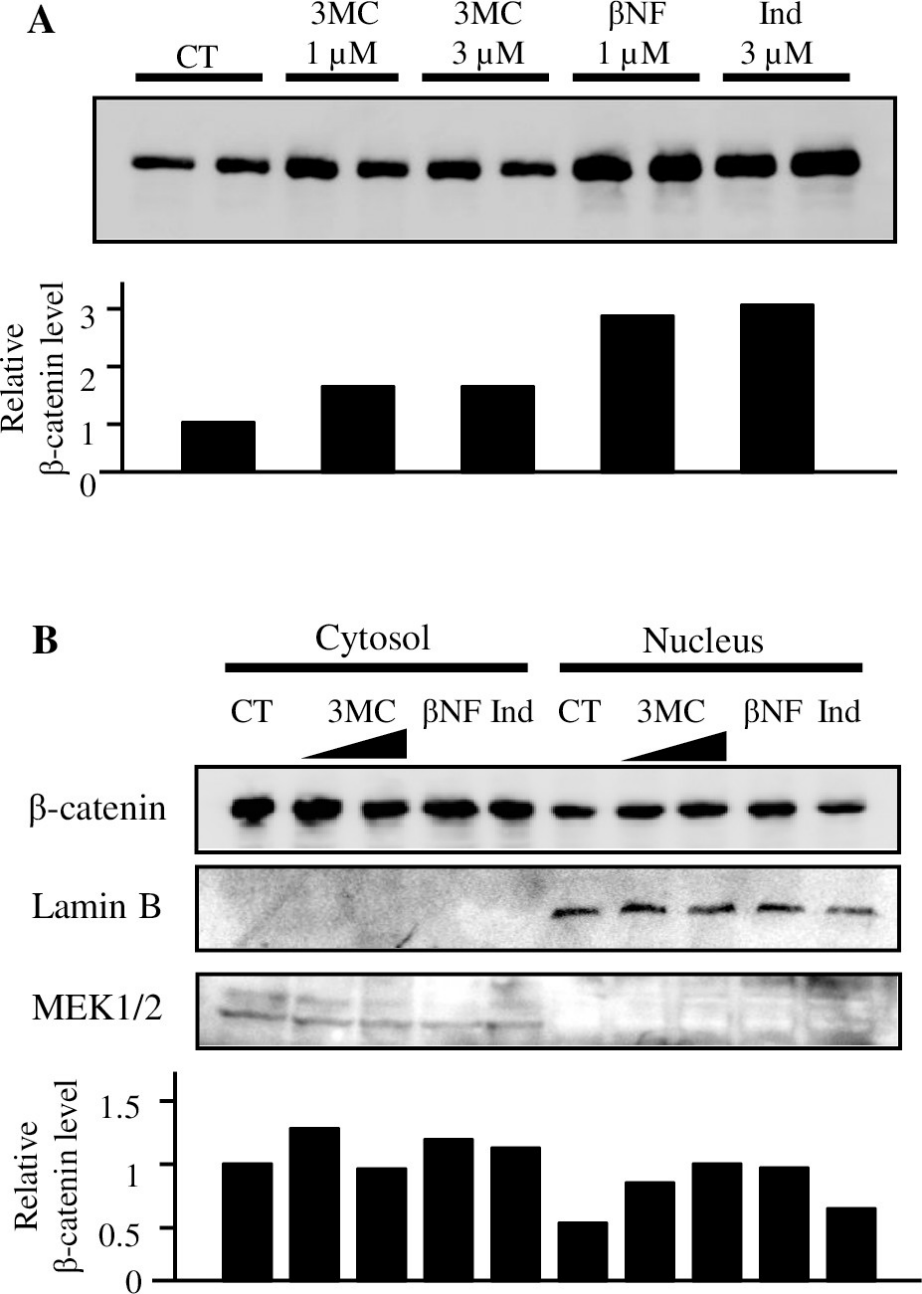
Change in β-catenin levels due to AhR ligand exposure in DLD-1 cells. **A**) DLD-1 cells were exposed to 3-methylcholanthrene (3MC; 1 or 3 µM), β-naphthoflavone (βNF; 1 µM), or indirubin (Ind; 3 µM) for 6 h. As a solvent control, 0.1% DMSO was added to cells (CT). Cytoplasmic proteins were extracted and 10 µg protein were subjected to western blotting with β-catenin antibodies. Individual band was quantitated in ImageQuant. Lower panel shows relative amount of β-catenin expression. Each bar represents the mean of duplicate determinations. **B**) Nuclear and cytoplasmic proteins were extracted from DLD-1 cells after 3MC (from left to right, 1 or 3 µM), βNF (1 µM), or Ind (3 µM) exposure for 6 h. Anti-Lamin B and anti-MEK1/2 antibodies were used in the samples. Lower panel shows relative amount of β-catenin expression compared to control (cytosol). Each bar represents the mean of duplicate determinations.

**Fig 2:**
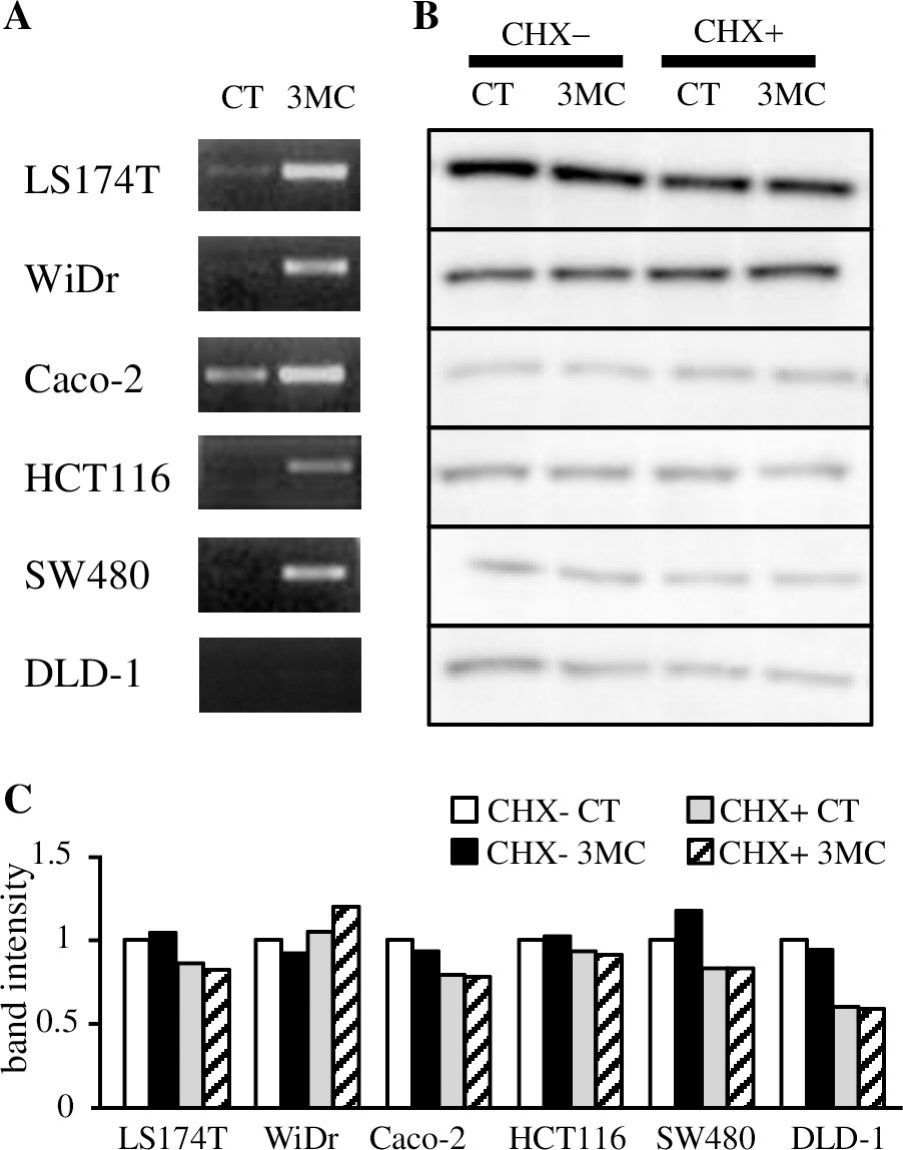
Change in β-catenin levels upon exposure of AhR ligands in various colon tumor cell lines. A) Induction of CYP1A1 mRNA by 3MC (1 μM) exposure in six colon tumor cell lines were investigated. After 6 hours of 3MC exposure, total RNA was isolated, and CYP1A1 mRNA was detected by semi-quantitative RT-PCR methods. The data showed amplified CYP1A1 cDNA in optimized cycles (24–36 cycles), which indicated marked differences. B) Six colon tumor cell lines were exposed to 3MC (1 µM) with or without 100 µM cycloheximide (CHX) serving as the solvent control (CT). After six-hour incubation, cell lysates were extracted and 10 or 20 µg of protein were subjected to western blotting with β-catenin antibodies. Representative blots are shown. C) Graph shows relative amount of β-catenin expression compared to control in each cell line. Bars represent the mean of duplicate determinations.

First, we confirmed whether AhR ligands can reduce cytoplasmic β-catenin protein levels in colorectal adenocarcinoma DLD-1 cells, which were used previously [16]. The efficacy of each ligand and its concentration were evaluated and shown in S1 Fig. AhR ligands, except IAA, showed about 3–5 fold induction of the XRE-dependent reporter gene. Regarding IAA exposure, the XRE-dependent reporter gene was not induced at 100 μM, and only a slight induction was shown at a concentration of 1 mM. As Fig 1A shows, cytoplasmic β-catenin protein levels were not reduced after AhR ligand exposure. The β-catenin levels exhibited an increase after β-naphthoflavone (βNF) or indirubin exposure. The degradation of β-catenin via AhR is thought to occur in the nucleus because CUL4B, a component of the AhR-associated ubiquitin ligase complex and known to locate in the nucleus [28], mediates the ubiquitination of β-catenin. We detected β-catenin from nuclear and cytoplasmic proteins separately following cotreatment with AhR ligands and cycloheximide because *de novo* synthesis of β-catenin could result in the degradation of β-catenin, rendering it undetectable. The reduction of the β-catenin was not obtained in the nucleus protein as well as cytosol protein after ligand exposure (Fig 1B). We next examined other colorectal cancer cell lines, including those used in the previous studies [16, 21]. Five cell lines, except DLD-1, showed CYP1A1 mRNA induction, thereby indicating AhR activation following 3MC exposure (Fig 2A). In DLD-1 cells, no band associated with CYP1A1 mRNA was detected on PCR, even after up to 36 cycles. Cycloheximide treatment slightly reduced β-catenin expression in most cell lines. However, exposure to 3-methylcholanthrene did not further reduce β-catenin expression in any of the tested cell lines (Fig 2B and 2C). In summary, we could not confirm the AhR-mediated degradation of β-catenin by ligands in any cell lines, even when *de novo* synthesis was inhibited.

### AhR signaling does not attenuate transcriptional activation mediated by β-catenin

**Fig 3:**
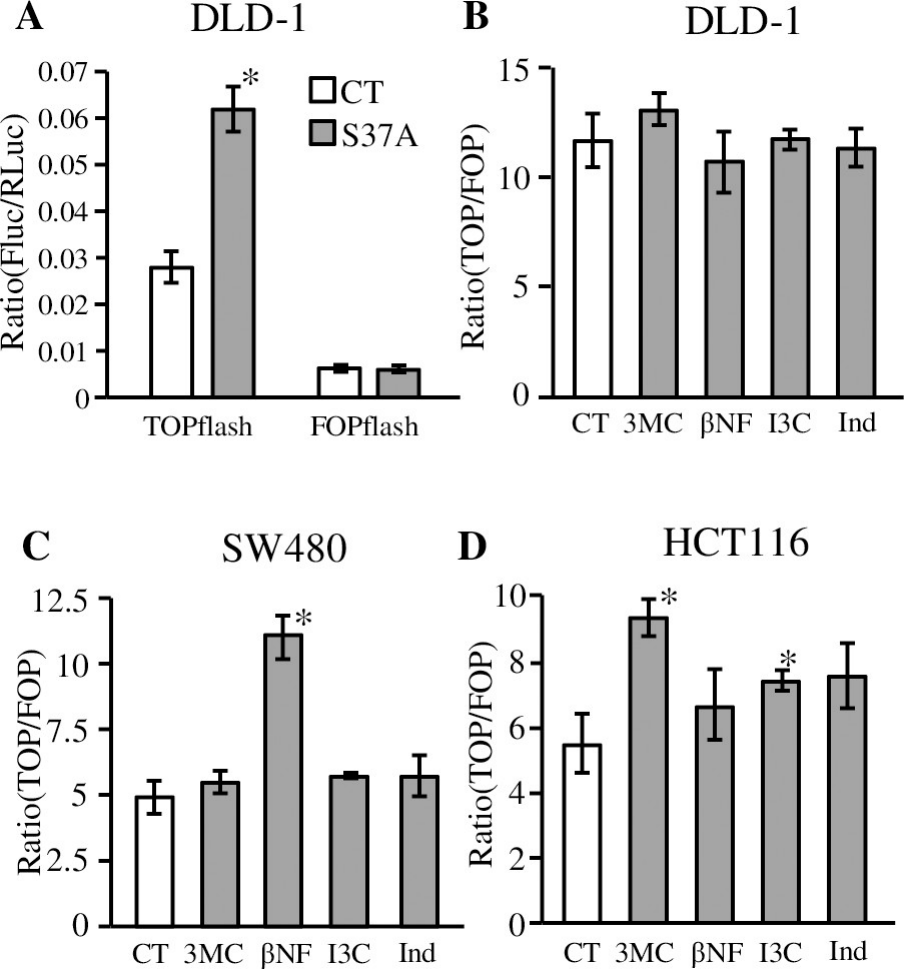
Effects on β-catenin/Tcf-dependent transcriptional activity upon exposure of various AhR ligands. **A**) DLD-1 cells were transfected with TOPflash or FOPflash reporter vectors and pRL-CMV together with empty vector (CT) or pCI-βcatS37A, the mutated β-catenin expression vector (S37A). The cells were lysed 48 hours after transfection, and luciferase activities were measured. Data represent the average of normalized firefly luciferase/*Renilla* luciferase activities of three independent experiments. **B-D**) TOPflash or FOPflash reporter vector was transfected into **B**) DLD-1 cells, **C**) SW480 cells, or **D**) HCT116 cells together with mutated β-catenin expression vector (S37A). After transfection, cells were exposed to 3MC (1 µM), βNF (1 µM), I3C (3 µM), Ind (3 µM), or 0.1% DMSO (solvent control, CT) for 8 hours. Data represent average of TOPflash/FOPflash ratios from three independent experiments. Statistically significant differences are denoted by asterisks (*p < 0.01).

Transactivation by β-catenin is thought to depend on the amount of activated β-catenin, which does not always correlate with the total amount of β-catenin protein. We investigated whether AhR signal attenuates transcriptional activation mediated by activated β-catenin using the TOPflash/FOPflash assay. A β-catenin-LEF/TCF sensitive (TOPflash) reporter vector or a β-catenin-LEF/TCF insensitive (FOPflash) reporter vector and expression vector of the hyperactive form of β-catenin (S37A mutant) were co-transfected into DLD-1 cells. As Fig 3A shows, S37A β-catenin mutant enhanced reporter gene transcription in the TOPflash vector (approximately 2-fold) but not in the FOPflash vector. We exposed various AhR ligands to DLD-1, SW480, and HCT116 cells after the TOPflash or FOPflash vector was transfected. All ligands failed to reduce TcF/LCF-dependent transcriptional activity, as shown in Fig 3B–3D. In SW480 cells, the exposure of βNF seemed to induce TcF/LCF-dependent transcriptional activity, which is represented as the TOPflash/FOPflash ratio. This was caused by decreased FOPflash value, which is unrelated to β-catenin, and the TOPflash value was unchanged. 3MC-treated HCT116 cells showed an increase in the TOPflash/FOPflash ratio, but other AhR ligands did not influence reporter gene expression. We concluded that the tested AhR ligands could not be reduced even though they could increase TcF/LCF-dependent transcriptional activity in these colorectal cancer cell lines.

### Verification of functional AhR expression in DLD-1 cells

**Fig 4:**
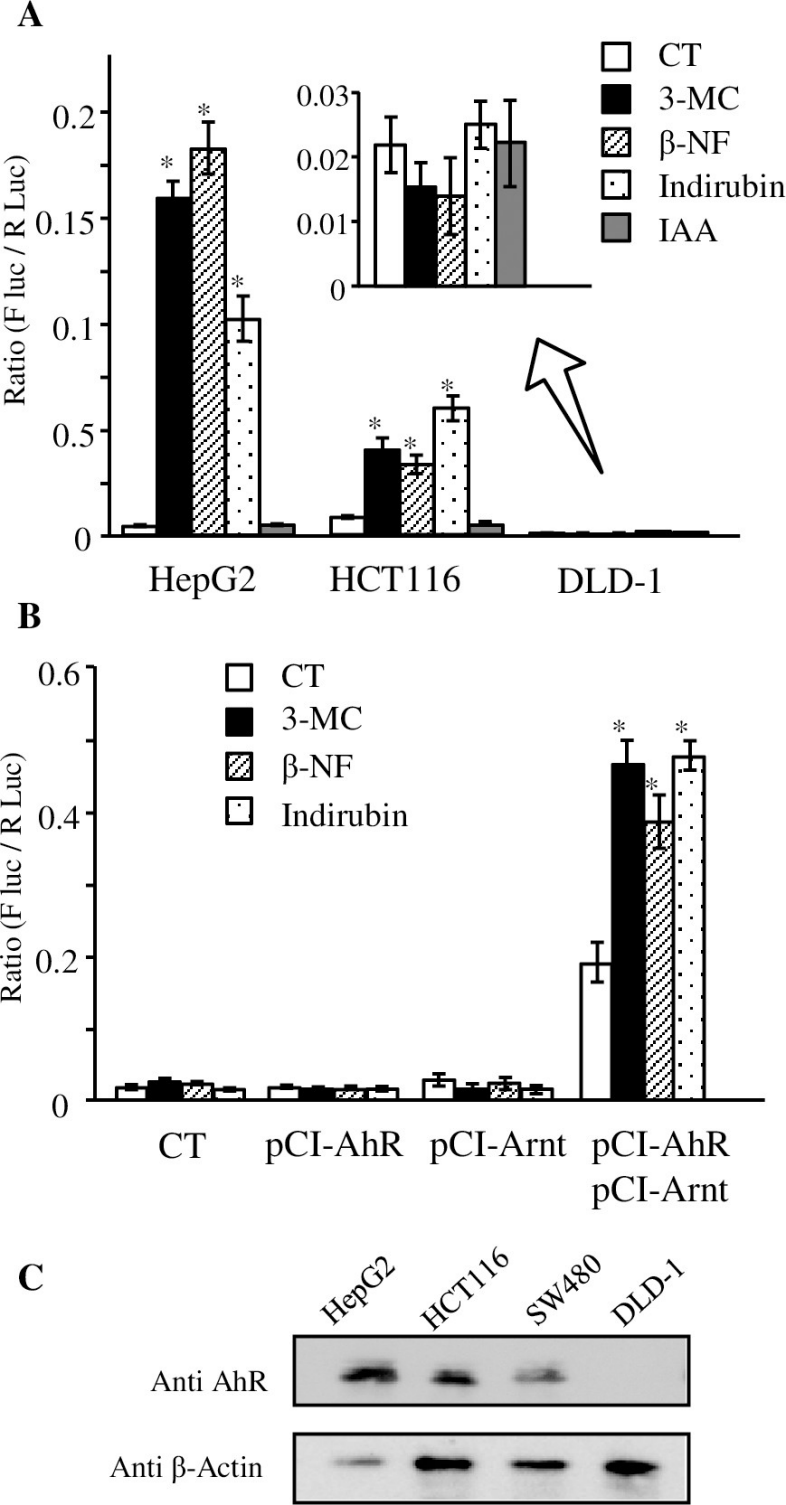
**A) Changes in AhR/Arnt mediated transcriptional activity in HepG2, HCT116, and DLD-1 cells.** Cells were transfected with XRE-dependent reporter vector pX4TK-Luc together with pRL-CMV vector. After transfection, cells were exposed to 3MC (1 µM), βNF (1 µM), indirubin (3 µM), IAA (100 µM), or DMSO (0.1%, solvent control, CT). The inset graph is data from DLD-1 cells with the enlarged scale. **B**) DLD-1 cells were transfected with pX4TK-Luc together with combinations of human AhR and human Arnt expression vector (pCI-AhR, pCI-Arnt). The mock plasmid, pCI-neo empty vector, was transfected instead of each expression vector as a negative control. After transfection, cells were exposed to 3MC (1 µM), βNF (1 µM), Ind (3 µM), or 0.1% DMSO (solvent control, CT) for 16 hours. Data represent the averages of normalized firefly luciferase/*Renilla* luciferase activities of three independent experiments. Statistically significant differences are denoted by asterisks (*p < 0.01, vs. control). **C**) AhR expression in HepG2, HCT116, and DLD-1 cells. Whole‐cell extracts were prepared in TNE buffer (10 mM Tris-HCl, pH 7.8; 1% NP-40; 150 mM NaCl; and 1 mM EDTA) with sonication. An aliquot of 10 µg (HepG2 cells) or 20 µg (HCT116 and DLD-1 cells) of cell lysates were subjected to western blotting with anti-AhR or anti- actin antibodies.

We evaluated ligand-dependent AhR activation in several colorectal cancer cell lines by CYP1A1 mRNA induction, as shown in Fig 2A. However, we obtained no amplification of CYP1A1 cDNA in DLD-1 cells even following 3MC exposure. Therefore, we examined whether DLD-1 cells express sufficient AhR and related factors. As Fig 4A shows, DLD-1 cells did not show XRE-dependent reporter gene induction. In contrast, HCT-116 cells and HepG2 cells responded to AhR ligands, except IAA. The experiment in which AhR and Arnt were additionally expressed revealed that DLD-1 cells require exogenous expression of these two proteins to respond to AhR ligands (Fig 4B). Additionally, immunoblot analysis detected AhR in HCT116 and SW480 cells, but not in DLD-1 cells (Fig 4C). We concluded that AhR and Arnt expression in DLD-1 cells must be insufficient. These data demonstrated that AhR-mediated β-catenin degradation is at least irreproducible in DLD-1 cells, despite the fact that this cell line was mainly used in previous reports.

### Alteration of β-catenin expression via AhR overexpression and knockdown

**Fig 5:**
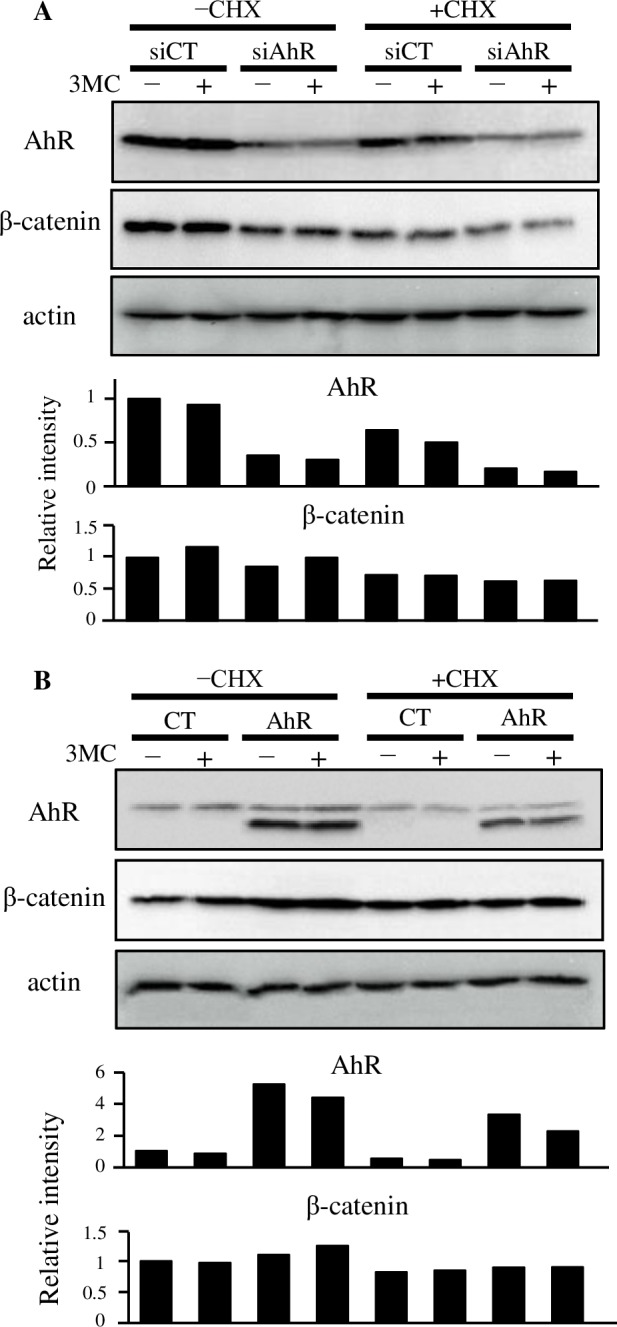
Alteration of β-catenin expression via AhR overexpression and knockdown. **A**) HCT116 cells were transfected with AhR (siAhR) or control (siCT) siRNA. Following 36 hours of transfection, cells were exposed to a combination of 3MC (1 µM) and CHX (100 µM), and whole cell lysates were extracted after 6 hours. **B**) Cells were transfected with a murine AhR expression vector pCI-mAhR or an empty vector pCI-neo. After 16 hours of transfection, cells were exposed to a combination of 3MC (1 µM) and CHX (100 µM), and whole cell lysates were extracted after 6 hours. Aliquots of the cell lysates containing 20 µg proteins were subjected to western blotting with AhR (upper panel), β-catenin (middle panel) or actin antibodies (lower panel). The relative intensity is shown in the bottom of the panel. The intensity of the AhR signal represents the sum of the values obtained from the two bands. The upper band corresponds to endogenous human AhR and the lower band corresponds to the overexpressed mouse AhR.

siRNA treatment reduced AhR expression to approximately 30% of the basal levels, resulting in slight downregulation of β-catenin expression compared with the control (Fig 5A, approximately 85%). Conversely, AhR overexpression in HCT116 cells (approximately 5-fold compared with controls) slightly increased β-catenin expression following 3MC treatment (Fig 5B). Both these alterations were restored by cycloheximide treatment (Fig 5A and 5B).

### Interaction between AhR and factors related to β-catenin degradation detected by yeast two-hybrid assays

**Fig 6:**
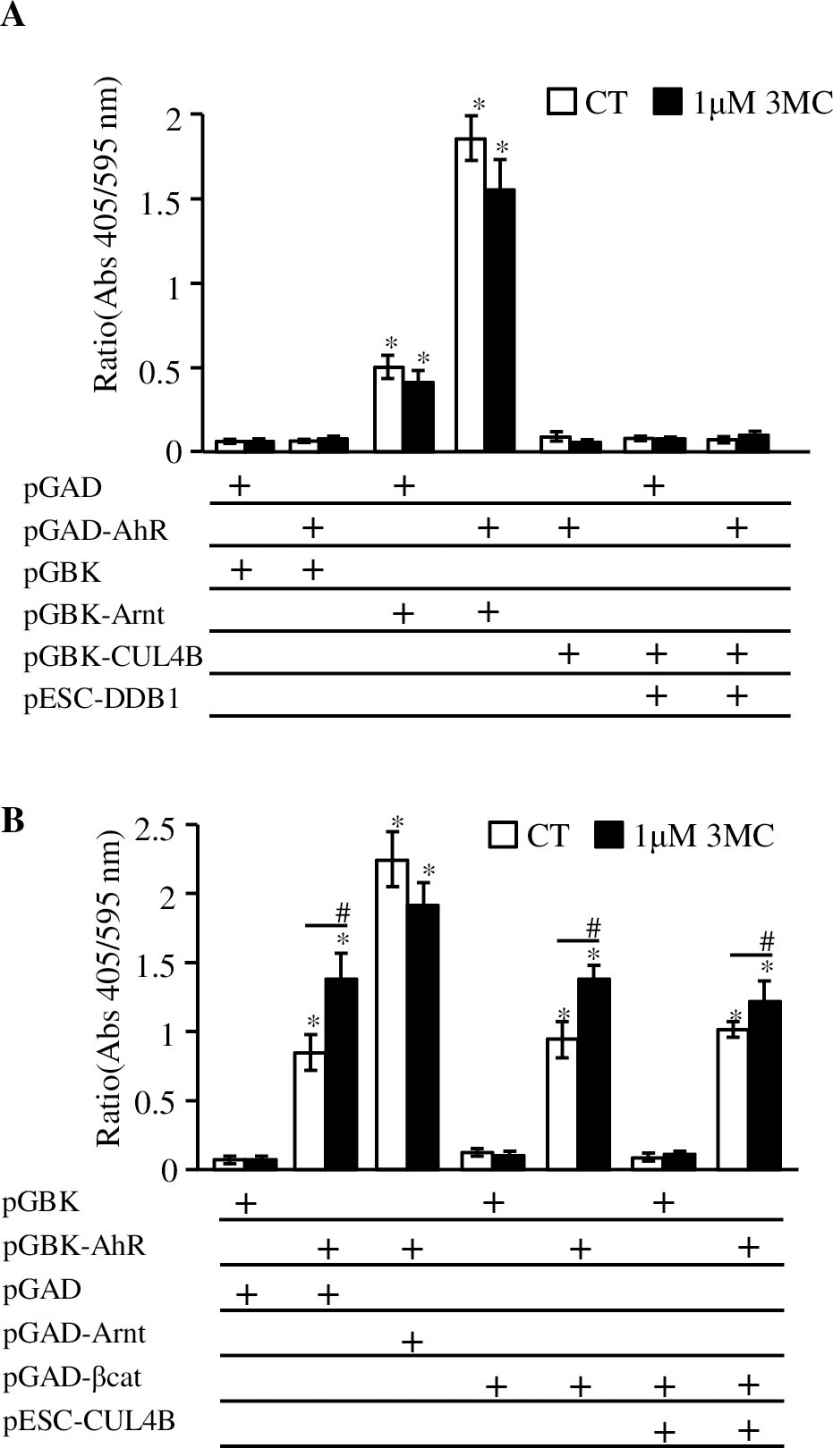
Yeast two-hybrid analysis failed to reconstitute the interaction between AhR and CUL4B. **A**) The plasmid vector pGADT7 containing the Gal4 activating domain and pGBKT7 containing Gal4 DNA-binding domain were co-expressed in yeast strain Y187. pGAD, parental empty vector pGADT7; pGAD-AhR, human AhR fused to Gal4 activating domain in pGADT7; pGBK, parental empty vector pGBKT7; pGBK-Arnt, human Arnt fused to Gal4 DNA-binding domain in pGBKT7; pGBK-CUL4B, human CUL4B fused to Gal4 DNA-binding domain in pGBKT7. The plasmid pESC-DDB1 is the vector expressing human DBD1 in yeast. **B**) The bait-prey exchange experiments for the interaction between AhR and CUL4B by yeast two-hybrid assay. pGBK-AhR, human AhR fused to Gal4 DNA-binding domain in pGBKT7; pGAD-Arnt, human Arnt fused to Gal4 activating domain in pGADT7; pGAD-βcat, human β-catenin fused to Gal4 activating domain in pGADT7; pESC-CUL4B is the vector expressing human CUL4B in yeast. Transformed yeast cells were exposed to 1 μM 3MC or 0.1% DMSO (solvent control, CT) for 12 h, and induced β-galactosidase activity was measured. The assays were performed in triplicate determinations, and values indicate the averages of the ratios of absorbances at 405 nm and 595 nm. Statistically significant differences are denoted by asterisks (*p < 0.01, vs. empty vectors, pGBK and pGAD; # *p* <0.01 (vs. solvent control).

We investigated protein-protein interactions by using the yeast two-hybrid assay. As Fig 6A shows, AhR fused with the Gal4 activating domain (pGAD-AhR) presented with no transcriptional activity. Meanwhile, Arnt fused to the Gal4 DNA-binding domain (pGBK-Arnt) showed significant induction of the reporter gene. When these hybrid proteins were co-expressed, transcriptional activity increased for about 3-fold compared with pGBK-Arnt alone, thus, reflecting the interaction of these proteins. CUL4B fused with the Gal4 activating domain (pGAD-CUL4B) showed no transcriptional alteration even in the presence of DDB1, the adaptor protein for CUL4B. In the experiment exchanging prey and bait proteins, AhR fused with the Gal4 DNA-binding domain (pGBK-AhR) showed some transcriptional activity as well as pGBK-Arnt (Fig 6B). 3MC exposure enhanced transcriptional activity, and the highest induction was obtained by co-expression of pGAD-Arnt. The fusion protein of CUL4B and the Gal4 activating domain (pGAD-CUL4B) did not affect the transcriptional activity of pGBK-AhR. Additional expression of DDB1 by co-transfection of pESC-DDB1 had no effect. These data indicated that the yeast two-hybrid assay could not reconstitute the interaction between AhR and CUL4B or AhR and β-catenin.

### Detection of the interaction between AhR and β-catenin in mammalian cells

**Fig 7:**
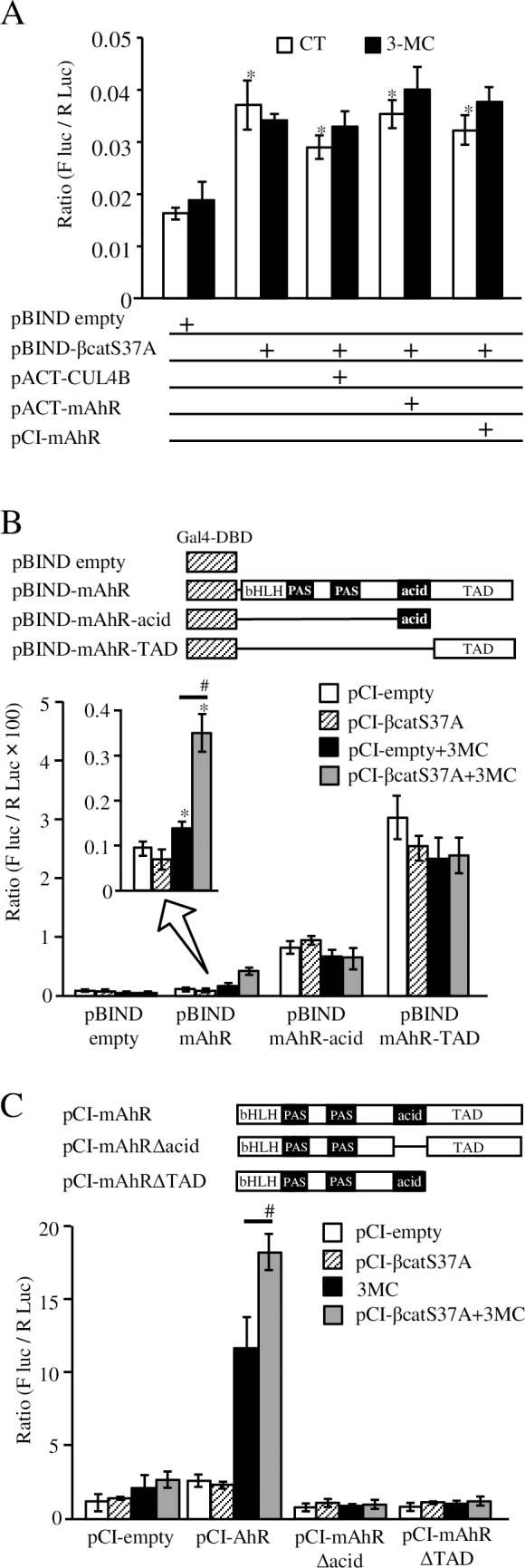
Interaction of AhR and β-catenin in mammalian cells detected by reporter gene assay. **A**) Detection of AhR and β-catenin interaction by the mammalian two-hybrid assay. The S37A β-catenin mutant cDNA was fused to Gal4 DNA-binding domain contained in the plasmid vector pBIND (pBIND-βcatS37A). Human CUL4B cDNA and mouse AhR cDNA were fused to VP16 activation domain in pACT vector, respectively (pACT-CUL4B and pACT-mAhR). pBIND empty is the parental empty vector. pCI-mAhR is the expression vector of mouse AhR in mammalian cells. These plasmids were co-transfected into HeLa cells with the reporter vector pG5Luc containing the luciferase gene under the control of the GAL4 response element. After transfection, cells were exposed to 3MC (1 µM) or DMSO (0.1%, solvent control CT) for 16 h. The firefly luciferase activity was normalized by *Renilla* luciferase activity expressed from the pRL-CMV vector. Data represent the averages of firefly luciferase activity normalized to *Renilla* luciferase activity expressed from experimental triplicates. **B**) Various mouse AhR cDNA fragments were fused to the Gal4 DNA-binding domain in the plasmid vector pBIND. Upper panel indicates scheme of each hybrid construct. bHLH: basic-helix-loop-helix DNA-binding domain, PAS: Per-Arnt-Sim domain containing ligand binding domain, acid: c-terminal acidic domain, and TAD: trans-activating domain. Plasmid vectors pBIND-mAhR, pBIND-mAhR-acid, and pBIND-mAhR-TAD contain full length cDNA, acidic domain (aa 524–583), and trans-activating domain (aa 425–805) of mouse AhR, respectively. Each of these plasmids and reporter vector pG5luc were transfected into HeLa cells together with pCI-βcatS37A, an expression vector for the S37A β-catenin mutant (β-catenin) or pCI-empty vector. After transfection, cells were exposed to 1 μM of 3MC, and luciferase activity was measured as described above. Data represent the averages of experimental triplicates. Statistically significant differences are denoted by asterisks (*p < 0.01, vs. pCI-empty; #*p* < 0.01, vs. without pCI-βcatS37A) **C**) β-Catenin acts as an enhancer of AhR in mammalian cells. AhR-dependent transcription and its enhancement by β-catenin require full length AhR. Upper panel indicates scheme of deleted AhRs. Mouse AhR cDNA fragments lacking acidic domain (pCI-mAhRΔacid: Δ aa 524–583) or c-terminal trans-activating domain (pCI-mAhRΔTAD: Δ aa 424–805) were transfected together with pCI-S37-β-catenin (S37-β-catenin), *Renilla* luciferase expression vector pRL-CMV, and reporter vector pX4TK-Luc. After transfection, cells were exposed to 3MC (1 µM) or DMSO (0.1%, solvent control CT) for 16 h. The firefly luciferase activity was normalized by *Renilla* luciferase activity expressed from the pRL-CMV vector. Data represent the averages of firefly luciferase activity normalized to *Renilla* luciferase activity expressed from experimental triplicates. Statistically significant differences are denoted by asterisks (#*p* < 0.01, vs. without pCI-βcatS37A).

Next, we investigated the interaction of β-catenin and AhR or CUL4B by using the mammalian two-hybrid assay. The S37A mutant of β-catenin fused with the GAL4 DNA-binding domain (pBIND-βcatS37A) in HeLa cells induced reporter gene activity (Fig 7A). AhR or CUL4B fused with the VP16 activation domain did not increase reporter gene activity via expression of pBIND-βcatS37A in either the presence or absence of the ligand. These data were consistent with our yeast two-hybrid assay results.

### Effect of β-catenin on transcriptional activity of AhR

In the experiment with exchanging prey and bait protein, full length mouse AhR fused to the Gal4 DNA-binding domain (pBIND-mAhR) showed ligand-dependent induction of the reporter gene (Fig 7B). Co-expression of the S37A β-catenin mutant enhanced the induction of the reporter gene in the presence of 3MC. The chimeric mouse AhR expressed from pBIND-mAhR-acid and pBIND-mAhR-TAD contained the acidic domain (aa 425–805) and the transactivation domain (aa 524–583), respectively, indicated higher reporter gene induction rather than the wild type mouse AhR. However, no alteration was observed by co-expression of the S37A β-catenin mutant (Fig 7B). To confirm whether this assay system reflects the binding of AhR and other proteins like coactivators, a similar experiment was performed with human steroid coactivator-1 (SRC-1), a typical nuclear receptor coactivator, instead of β-catenin [29]. Unlike β-catenin, SRC-1 enhanced transcriptional activity by binding to the transactivation domain of AhR but not to the acidic domain alone (S2 Fig).

Since the acidic domain fused to Gal4-DBD (pBIND-mAhR-acid) seemed to have transcriptional activation function, we constructed AhR lacking the acidic domain (pCI-mAhRΔacid) or transcriptional activation domain (pCI-mAhRΔTAD) as outlined previously (see supplemental information in [15]). The XRE-dependent reporter assay showed that co-expression of hyperactive β-catenin significantly enhanced AhR-mediated transcriptional activity in a ligand dependent manner (Fig 7C). The lack of the acidic domain as well as transcriptional activation domain in AhR completely abolished transcriptional activity regardless of β-catenin expression.

Although the transactivation and acidic domains of AhR are responsible for the enhancement of AhR-mediated transactivation via coactivator binding, each of these domains alone is insufficient for the enhancement of transactivation by β-catenin.

### Detection of the interaction between AhR and β-catenin by immunoprecipitation

**Fig 8:**
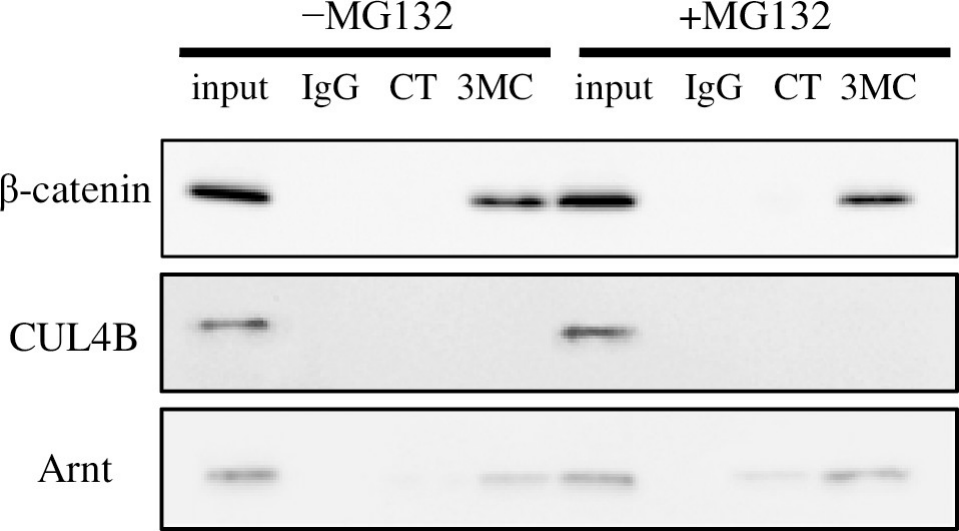
Coimmunoprecipitation of β-catenin or CUL4B with an AhR antibody. Wild-type β-catenin expression vectors pCI-βcat and pCI-mAhR were cotransfected into HCT116 cells, and the transfected cells were exposed to 1 µM 3MC (3MC) or DMSO (CT, solvent control) with or without 100 μM MG-132 for 2 h. Nuclear lysates were extracted and coimmunoprecipitated by AhR-specific antibodies. Immunoprecipitated fractions were analyzed by SDS–PAGE and immunoblotting using specific antibodies for β-catenin (upper panel), CUL4B (middle panel), or Arnt (lower panel). Input (Lane 1 and 5) represents 1/20 of the lysates from cells treated with 3MC presence or absence of MG-132. As a negative control, immunoprecipitation was performed using control (non-specific) IgG (lane2 and 6, IgG).

Several reports have investigated β-catenin and AhR interaction by co-immunoprecipitation methods, presenting conflicting data illustrating both detection and non-detection. By using specific antibodies against AhR and β-catenin, we detected a complex of these proteins from HCT116 cells after transfection of pCI-mAhR and pCI-βcat (Fig 8). β-Catenin was detected in nuclear proteins prepared from 3MC-treated cells following coimmunoprecipitation with specific antibodies against AhR (Fig 8). MG-132 treatment did not alter the intensity of the positive β-catenin band. In contrast, CUL4B was not detectable even though β-catenin and AhR were overexpressed. These data indicate the formation of a complex between β-catenin and ligand-activated AhR in the nucleus.

## Discussion

Protein-protein interactions are essential for regulating most cellular functions. Among an enormous number of combinations between proteins, protein interactions involving β-catenin are recognized to be particularly important for colorectal cancer. AhR was reported as an interacting protein with β-catenin, but studies dissecting their crosstalk have generated conflicting outcomes. In this study, we focused on ligand-dependent β-catenin degradation via AhR, and we attempted to verify previous data about AhR and β-catenin interactions. In conclusion, we found several inconsistencies in results reported previously, such as studies illustrating that AhR acts as an ubiquitin ligase and degrades β-catenin.

Previous results exhibiting decreased β-catenin expression were not recapitulated in our study using colorectal cancer cell lines, even when *de novo* synthesis was inhibited (Fig 2B). The TOPflash assay indicated transcriptional activity related to β-catenin activation. The reduction of TOPflash reporter gene activity via the exposure of AhR ligands was not seen in DLD-1, SW480, and HCT116 cells (Fig 3). As Fig 4 shows, DLD-1 cells do not express sufficient AhR and Arnt. Because cross contamination with HCT-15 cells has been suspected regarding the origin of the DLD-1 cell line [30], we performed additional experiments by using newly purchased DLD-1 cells from another distributor, but the results were unchanged (S3 Fig). These results represent a major contradiction with previous reports elucidating the mechanism of β-catenin degradation via AhR, which was primarily demonstrated using DLD-1 cells [16]. Besides, HCT116 and SW480 cell lines expressed AhR (Fig 4C) and indicated responses to AhR ligands, except IAA (Fig 4A). However, these cell lines did not illustrate reduced β-catenin protein levels nor suppressed TOP flash reporter activity. In addition, AhR overexpression and knockdown resulted in slight alterations of β-catenin. These contradictory data cannot be explained by our current results. However, since these alterations were recovered by cycloheximide treatment, cellular AhR levels may affect β-catenin expression. However, these results do not appear to reflect β-catenin degradation via AhR.

We concluded that it would be highly difficult to reproduce previous in vitro experiments elucidating the mechanisms of β-catenin degradation via AhR [16]. A recent study using the colon carcinoma cell line Caco-2 reported that β-catenin was not reduced after TCDD exposure, and AhR agonistic activity of IAA was only apparent at a higher dose (0.5–1 mM) [22]. Our data are consistent with this study.

We tried to define AhR and β-catenin or AhR and CUL4B interactions by using the yeast two-hybrid assay because previous studies have clearly demonstrated interaction between β-catenin and the FIP200 protein [31]. However, we could not obtain any evidence that AhR binds to either β-catenin or CUL4B (Fig 6A and 6B). However, the binding between these proteins in mammalian cells may not be reproducible in yeast as the required proteins are absent. The yeast strain *S*. *cerevisiae* Y184 used in this experiment has orthologs of several factors required for the ubiquitin ligase activity of CUL4B: UTP13 for TBL3 and Ddb1 for DDB1. However, it is unclear whether these yeast orthologs were functional in the E3 ubiquitin ligase complex, including human CUL4B. Thus, the indirect interaction between AhR and β-catenin mediated by CUL4B-containing complexes is thought to be reconstituted only in mammalian cells. By using the mammalian two-hybrid assay, GAL4-DBD fused with β-catenin did not interact with either AhR or CUL4B in HeLa cells (Fig 7A). Meanwhile, in the experiment with exchanging prey and bait proteins, β-catenin enhanced transcriptional activity of full length AhR fused to GAL4-DBD in a ligand dependent manner (Fig 7B). The data allowed for two interpretations: there was binding between AhR and β-catenin that occurs prior to ubiquitination and degradation, or these data indicated enhancer activity of β-catenin for AhR-dependent transcription. Therefore, we have constructed several deletion mutants and chimeric proteins, including mouse AhR lacking the acidic domain (AhRΔacid in Fig 7C) as per a previous report (See supplemental information in [15]). The mouse AhR lacking the acidic domain has been reported to have transactivation function but lacks CUL4B binding capability. Distinct from the results in the previous report, the mutant mouse AhR lacking the acidic domain did not show any transcriptional activity in our experiments. We also performed similar experiments using MCF-7 cells to be consistent with the previous report, and we could not detect any additional transcriptional activity via the expression of mouse AhR lacking the acidic domain (S4 Fig). Moreover, chimeric AhR with the Gal4-DNA-binding domain elucidated that the acidic domain of mouse AhR showed transcriptional activation function (Fig 7B). We concluded that this domain is an essential part of the transactivation domain of AhR and it alone cannot mediate the enhancer activity of β-catenin. The requirement of full length AhR for the enhancer activity of β-catenin indicated interaction between AhR and β-catenin would not be so simple, unlike SRC-1 which only requires a subdomain for its binding (S2 Fig). Further investigation is warranted to elucidate the enhancement of β-catenin-mediated AhR signaling. These data confirmed the enhancer activity of β-catenin in AhR signaling as gleaned in previous studies [12–13].

The binding of β-catenin to AhR might require other related proteins in mammalian cells. Therefore, we attempted to detect the interaction of AhR and β-catenin in HCT116 cells via coimmunoprecipitation (Fig 8). A previous study detected β-catenin in Caco-2 cells following coimmunoprecipitation with an AhR antibody [21]. However, in that report, immunoprecipitation with an antibody against β-catenin followed by AhR detection failed to demonstrate the interaction between AhR and β-catenin. In the present study, we detected β-catenin from nuclear proteins via communoprecipitation assays following the same series of steps as described previously [21]. The HCT116 cell line is heterozygous for the β-catenin gene, harboring one wild-type and one mutant allele, with a deletion of Ser45, which is phosphorylated by GSK3β [32]. Thus, wild-type mouse β-catenin was overexpressed in order to be dominant over mutant β-catenin in HCT116 cells. In addition, effects of the proteasome inhibitor MG-132 on β-catenin levels were investigated since proteasome degradation may not detect the interactions between AhR and β-catenin. The nuclear distribution of CUL4B has been reported [33], with the interaction between β-catenin and AhR occurring in the nucleus, and β-catenin has been thought to be degraded via nuclear proteasomes. Therefore, if β-catenin is indeed degraded via nuclear proteasome, MG-132 treatment is expected to promote increased β-catenin and AhR binding. However, the level of nuclear β-catenin interacting with AhR was not altered by MG-132 treatment. In addition, there was no interaction between AhR and CUL4B under any condition tested. Collectively, these data suggest a nuclear interaction between AhR and β-catenin that is associated with enhanced AhR signaling but may not lead to β-catenin degradation.

Results obtained from our yeast two-hybrid and coimmunoprecipitation assays did not indicate any interaction between AhR and CUL4B. Tripathi and colleagues reported physical interactions between CUL4B and β-catenin in *Drosophila* [34]. They also immunoprecipitated CUL4B from PCC4, C3H T10 (1/2), and HeLa cells, and they succeeded in detecting β-catenin. It was reported that the N-terminal domain (1–191) of CUL4B is essential to binding with AhR due to subsequent ubiquitination of the target protein, including β-catenin [15]. However, amino acid homology between *Drosophila* and human CUL4B at this region is only 16%. Therefore, even if there was any AhR present to inhibit the interaction between β-catenin and CUL4B in human cells, it would not be similar to the interaction observed in *Drosophila*.

A recent detailed study on the interaction between AhR and CUL4B using human cell lines presented conflicting data compared to work by Ohtake and colleagues [15]. Luecke-Johansson and colleagues could not identify Arnt as a part of the CUL4B/AhR complex when performing co-immunoprecipitations with a CUL4B antibody, even after treatment with TCDD, although Arnt had been shown to be essential for the binding between AhR and CUL4B in the previous report [35]. Moreover, a recent study showed that over-expression of CUL4B increased β-catenin accumulation. The report illustrated that CUL4B activated Wnt/β-catenin signaling by protecting β-catenin from GSK3-mediated degradation and promoted proliferation of human osteosarcoma and hepatoma cells [36]. According to the report, the role of CUL4B to β-catenin is considered to be a positive regulator in these cells. Recently, many studies have reported a positive correlation between CUL4B expression and cellular β-catenin [37–39]; however, there have been no subsequent reports of the association of CUL4B with β-catenin degradation.

A previous report showing β-catenin degradation via AhR ligands included *in vivo* data showing that cecal tumors spontaneously developed in AhR-knockout mice [16]. In that report, histochemical analyses also showed the nuclear accumulation of β-catenin in small intestinal cells. Another study [40] following this report reconfirmed increased β-catenin expression within the cecum of AhR-knockout mice. Thus, AhR indeed contributes to β-catenin degradation *in vivo* even though β-catenin cannot be degraded *in vitro* by AhR-mediated ubiquitination. Our data only show that β-catenin degradation was not reproducible *in vitro*; thus, the association between β-catenin and AhR *in vivo* remains unclear.

Overall, we could only corroborate one-way interplay: β-catenin enhances AhR-mediated transcriptional activation. These results suggested that the degradation of β-catenin via ligand-bound AhR is irreproducible. This work will open up discussion regarding the conflicting data present in the field, which will warrant future studies to truly dissect the mechanisms governing this phenomenon.

## Supporting information

S1 Fig Evaluation of AhR agonist activity of various ligands. HCT116 cells were transfected with reporter genes pX4TK-Luc and pCMV-Rluc. Cells were exposed to 0.1% DMSO (CT, solvent control), 1 or 3 μM 3-methylcholanthrene (3MC), 1 μM β-naphthoflavone (βNF), 3 μM indirubin (Ind), 100 μM indole-3-carbinol (I3C), or 100 μM or 1 mM indole-3-acetate (IAA). After 16 h of incubation, the cells were lysed, and firefly and Renilla luciferase activities were measured. Data represent the average of normalized firefly luciferase/Renilla luciferase activities from experimental duplicates.
(PDF)

S2 Fig Binding of SRC-1 and mouse AhR subdomain. Upper panel indicates scheme of each hybrid construct. Various mouse AhR cDNA fragments were fused to the Gal4 DNA-binding domain in the plasmid vector pBIND. pBIND empty, parental empty vector; pBIND-mAhR, containing full length mouse AhR; pBIND-mAhR-TAD, containing mouse AhR trans-activating domain (aa 425–805); pBIND-mAhR-acid, containing mouse AhR c-terminal acidic domain (aa 524–583). Each of these plasmids and reporter vector pG5luc were transfected into HeLa cells together with pCI-SRC1, an expression vector for human SRC-1, or pCI-empty vector. After transfection, cells were exposed to 3MC (1 µM) or DMSO (0.1%, solvent control CT) for 16 h. Data represent the average of firefly luciferase activity normalized to Renilla luciferase activity expressed from experimental duplicates.
(PDF)

S3 Fig Loss of ligand-dependent transcriptional activity via AhR/Arnt in DLD-1 cells. An experiment similar to Fig 4B was performed to reconfirm the data using newly purchased DLD-1 cells from Health Science Research Resources Bank (HSRRB, Osaka, Japan; Lot # 09082004, Cell # JCRB9094). The cells were transfected with XRE-dependent reporter vector together with AhR and Arnt expression vectors. Mock plasmid, pCI-neo empty vector, was transfected as a negative control (empty). After transfection, cells were exposed to 0.1% DMSO (CT, solvent control), 1 μM 3-methylcholanthrene (3MC), 1 μM β-naphthoflavone (βNF), or 3 µM indirubin (Ind) for 16 h. Data represent the average of normalized firefly luciferase/Renilla luciferase activities of three independent experiments. The inset graph is the data from DLD1 cells with the enlarged scale. Statistically significant differences are denoted by asterisks (*p < 0.01, vs. control).
(PDF)

S4 Fig The acidic domain of AhR functioned as part of the transactivation domain in MCF-7 similar to the results obtained by HeLa cells (Fig 4). Upper panel indicates scheme of each hybrid construct. Plasmid vector containing mouse AhR cDNA (pCI-mRNA), lacking acidic domain (pCI-mAhRΔacid: Δ aa 524–583) or c-terminal trans-activating domain (pCI-mAhRΔTAD: Δ aa 424–805) were transfected together with Renilla luciferase expression vector pRL-CMV and reporter vector pX4TK-Luc into MCF-7 cells. Mock plasmid, pCI-empty vector, is the parental plasmid without cDNA insertion. After transfection, cells were exposed to 1 μM 3MC, and luciferase activity was measured. Data represent the average of experimental duplicates.
(PDF)

S1 Table Primers used for plasmid construction. (DOCX)

S1 File Raw data shown in Figs 1–7. (XLSX)
