# Supplementary figures and images for: Insight into the relationship between aryl-hydrocarbon receptor and β-catenin in human colon cancer cells
Source: PLoS One. 2019 Nov 1;14(11):e0224613. doi: 10.1371/journal.pone.0224613 (PMC6824560; doi:10.1371/journal.pone.0224613)

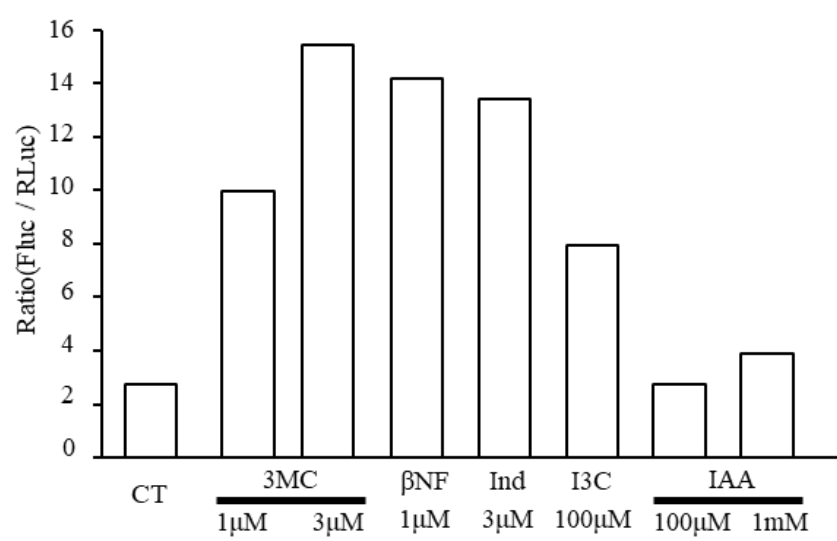

Supplement: S1 Fig — HCT116 cells were transfected with reporter genes pX4TK-Luc and pCMV-Rluc. Cells were exposed to 0.1% DMSO (CT, solvent control), 1 or 3 μM 3-methylcholanthrene (3MC), 1 μM β-naphthoflavone (βNF), 3 μM indirubin (Ind), 100 μM indole-3-carbinol (I3C), or 100 μM or 1 mM indole-3-acetate (IAA). After 16 h of incubation, the cells were lysed, and firefly and Renilla luciferase activities were measured. Data represent the average of normalized firefly luciferase/Renilla luciferase activities from experimental duplicates. (PDF) [file pone.0224613.s001.pdf]

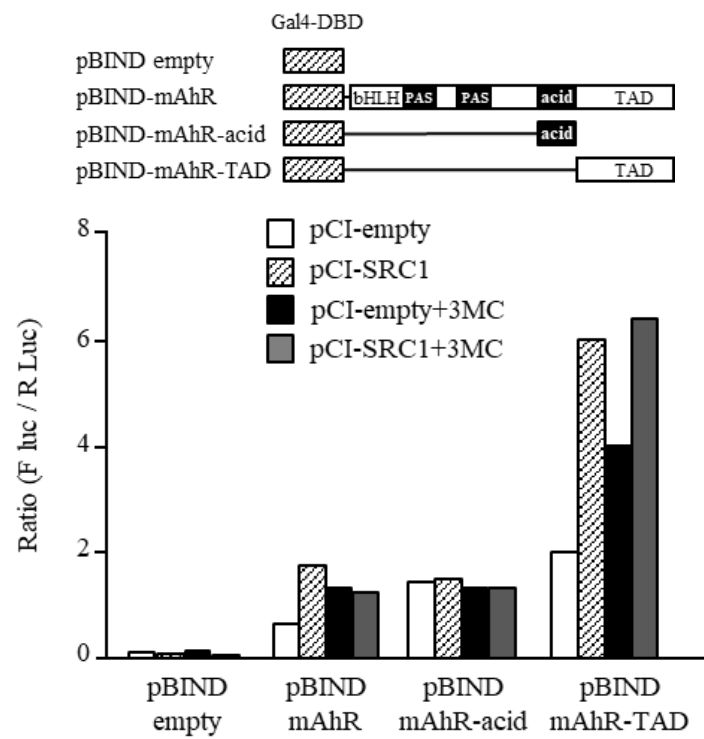

Supplement: S2 Fig — Upper panel indicates scheme of each hybrid construct. Various mouse AhR cDNA fragments were fused to the Gal4 DNA-binding domain in the plasmid vector pBIND. pBIND empty, parental empty vector; pBIND-mAhR, containing full length mouse AhR; pBIND-mAhR-TAD, containing mouse AhR trans-activating domain (aa 425–805); pBIND-mAhR-acid, containing mouse AhR c-terminal acidic domain (aa 524–583). Each of these plasmids and reporter vector pG5luc were transfected into HeLa cells together with pCI-SRC1, an expression vector for human SRC-1, or pCI-empty vector. After transfection, cells were exposed to 3MC (1 µM) or DMSO (0.1%, solvent control CT) for 16 h. Data represent the average of firefly luciferase activity normalized to Renilla luciferase activity expressed from experimental duplicates. (PDF) [file pone.0224613.s002.pdf]

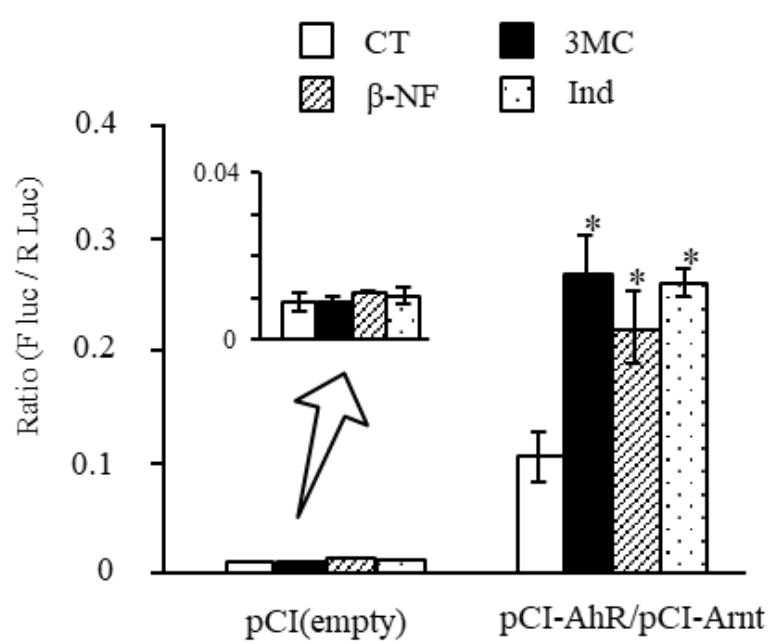

Supplement: S3 Fig — An experiment similar to Fig 4B was performed to reconfirm the data using newly purchased DLD-1 cells from Health Science Research Resources Bank (HSRRB, Osaka, Japan; Lot # 09082004, Cell # JCRB9094). The cells were transfected with XRE-dependent reporter vector together with AhR and Arnt expression vectors. Mock plasmid, pCI-neo empty vector, was transfected as a negative control (empty). After transfection, cells were exposed to 0.1% DMSO (CT, solvent control), 1 μM 3-methylcholanthrene (3MC), 1 μM β-naphthoflavone (βNF), or 3 µM indirubin (Ind) for 16 h. Data represent the average of normalized firefly luciferase/Renilla luciferase activities of three independent experiments. The inset graph is the data from DLD1 cells with the enlarged scale. Statistically significant differences are denoted by asterisks (*p < 0.01, vs. control). (PDF) [file pone.0224613.s003.pdf]

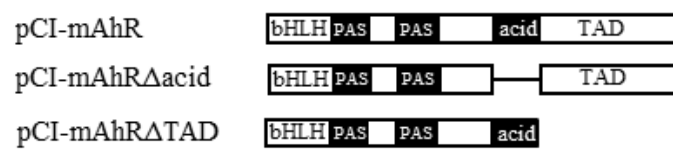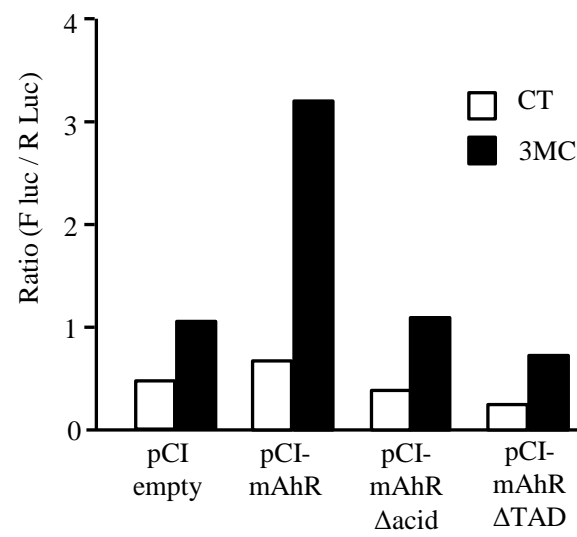

Supplement: S4 Fig — Upper panel indicates scheme of each hybrid construct. Plasmid vector containing mouse AhR cDNA (pCI-mRNA), lacking acidic domain (pCI-mAhRΔacid: Δ aa 524–583) or c-terminal trans-activating domain (pCI-mAhRΔTAD: Δ aa 424–805) were transfected together with Renilla luciferase expression vector pRL-CMV and reporter vector pX4TK-Luc into MCF-7 cells. Mock plasmid, pCI-empty vector, is the parental plasmid without cDNA insertion. After transfection, cells were exposed to 1 μM 3MC, and luciferase activity was measured. Data represent the average of experimental duplicates. (PDF) [file pone.0224613.s004.pdf]
