# Supplementary material for: Insight into the relationship between aryl-hydrocarbon receptor and β-catenin in human colon cancer cells
Source: PLoS One. 2019 Nov 1;14(11):e0224613. doi: 10.1371/journal.pone.0224613 (PMC6824560; doi:10.1371/journal.pone.0224613)
Supplement: S1 Table — (DOCX) [file pone.0224613.s005.docx]

Table S1. Primers used for plasmid construction.

|  | plasmid name | primer sequence |
| --- | --- | --- |
| plasmid vectors for yeast two-hybrid assay | | |
|  | pGAD-T7 and pGBK-T7* | |
|  |  | 5'-CATGGAGGCCGAATTCCCGGGGATCCG-3' |
|  |  | 5'-TCGACGGATCCCCGGGAATTCGGCCTC-3' |
|  | pGAD-AhR and pGBK-AhR | |
|  |  | 5'-ATACCTCCATGGACAGCAGCAGCGCCAACA-3' |
|  |  | 5'-AATTGAGATCTTACAGGAATCCACTGGATG-3' |
|  | pGAD-Arnt and pGBK-Arnt | |
|  |  | 5'-CATCTGGATCCATGGCGGCGACTACTGCCAACC-3' |
|  |  | 5'-ACCCCTCGAGTTCTATTCTGAAAAGGGGGGAAAC-3' |
|  | pGAD-βcat |  |
|  |  | 5'-TATACCCGGGGACAATGGCTACTCAAGCTG-3' |
|  |  | 5'-TAAGTCGACATTTACAGGTCAGTATCAAAC-3' |
|  | pGBK-CUL4B |  |
|  |  | 5'-ACCCAGGATCCGGATGATGTCACAGTCATC-3' |
|  |  | 5'-GCAAGCTCGAGATTCTATGCAATATAGTTG-3' |
| plasmid vectors for yeast expression | | |
|  | pESC-CUL4B |  |
|  |  | 5'-ACCCAGGATCCGGATGATGTCACAGTCATC-3' |
|  |  | 5'-GCAAGCTCGAGATTCTATGCAATATAGTTG-3' |
|  | pESC-DDB1 |  |
|  |  | 5'-GCTCCAAGATCTACATGTCGTACAACTACG-3' |
|  |  | 5'-CCCTGCCGTCGACTAATGGATCCGAGTTAG-3' |
| plasmid vectors for mammalian two-hybrid assay | | |
|  | pACT-mAhR |  |
|  |  | 5'-AACTCGAGATGAGCAGCGGCGCCAACATCACCTA-3' |
|  |  | 5'-AAGGTCTAGATCAACTCTGCACCTTGCTTAGG-3' |
|  | pACT-CUL4B |  |
|  |  | 5'-GACGACCCAAAGGACGGATGATGT-3' |
|  |  | 5'-GCAAGCTCGAGATTCTATGCAATATAGTTG-3' |
|  |  | 5'-GCTGCAAGGCCAACATTCTATGC-3' |
|  |  | 5'-ACCCAGGATCCGGATGATGTCACAGTCATC-3' |
|  | pBIND-mAhR |  |
|  |  | 5'-AACTCGAGATGAGCAGCGGCGCCAACATCACCTA-3' |
|  |  | 5'-AAGGTCTAGATCAACTCTGCACCTTGCTTAGG-3' |
|  | pBIND-mAhR-acid |  |
|  |  | 5'-AAGGTCTAGATCACAGGGAATCCTGCACGTAGG-3' |
|  |  | 5'-TTCCACGCGTTACGCACCAAAAGCAACACT-3' |
|  | pBIND-mAhR-TAD |  |
|  |  | 5'-TTCCACGCGTTACGCACCAAAAGCAACACT-3' |
|  |  | 5'-AAGGTCTAGATCAACTCTGCACCTTGCTTAGG-3' |
|  | pBIND-βcatS37A |  |
|  |  | 5'-TATACCCGGGGACAATGGCTACTCAAGCTG-3' |
|  |  | 5'-TAAATCTAGATTTACAGGTCAGTATCAAAC-3' |
| plasmid vectors for mammalian expression | | |
|  | pCI-mAhRΔacid: ΔA.A.524-583 | |
|  |  | 5'-TTCCACGCGTTGAACAATTCAACTTTGCTG-3' |
|  |  | 5'-GGACGCGTTCAACTCTGCACCTTGCTTAGGAATG-3' |
|  |  | 5'-AAAGAGCTCTGAGGGGCCGCCAGAGAGTGC-3' |
|  |  | 5'-AAGGTCTAGATCAGCGTATTGGTAGGGGATCC-3' |
|  | pCI-mAhRΔTAD: ΔA.A.424-805 | |
|  |  | 5'-AACTCGAGATGAGCAGCGGCGCCAACATCACCTA-3' |
|  | pCI-βcat S37A |  |
|  |  | 5'-CTCTGGAATCCATGCTGGTGCCACTACCAC-3' |
|  |  | 5'-GTGGTAGTGGCACCAGCATGGATTCCAGAG-3' |

*oligonucleotides for modification of multi-cloning sites in pGAD-T7 and pGBK-T7
